# Quantum Biology on the Edge of Quantum Chaos

**DOI:** 10.1371/journal.pone.0089017

**Published:** 2014-03-06

**Authors:** Gabor Vattay, Stuart Kauffman, Samuli Niiranen

**Affiliations:** 1 Department of Physics of Complex Systems, Eotvos University, Budapest, Hungary; 2 Vermont Complex Systems Center, University of Vermont, Burlington, Vermont, United States of America; 3 Department of Signal Processing, Tampere University of Technology, Tampere, Finland; University of California, Merced, United States of America

## Abstract

We give a new explanation for why some biological systems can stay quantum coherent for a long time at room temperature, one of the fundamental puzzles of quantum biology. We show that systems with the right level of complexity between chaos and regularity can increase their coherence time by orders of magnitude. Systems near Critical Quantum Chaos or Metal-Insulator Transition (MIT) can have long coherence times and coherent transport at the same time. The new theory tested in a realistic light harvesting system model can reproduce the scaling of critical fluctuations reported in recent experiments. Scaling of return probability in the FMO light harvesting complex shows the signs of universal return probability decay observed at critical MIT. The results may open up new possibilities to design low loss energy and information transport systems in this Poised Realm hovering reversibly between quantum coherence and classicality.

## Introduction

Discovery of room temperature quantum coherence in the avian compass [1] of birds, in the olfactory receptors [2] and in light harvesting complexes [3]–[6] in the last few years indicate that quantum effects might be ubiquitous in biological systems. While the quantum chemical understanding of the details of light harvesting systems is almost complete, no organizing principle has been found which could explain why quantum coherence is maintained in these systems for much longer than the characteristic decoherence time imposed by their room temperature environment. Here we propose that at the critical edge of quantum chaos coherence and transport can coexist for several orders of magnitudes longer than in simple quantum systems. Quantum systems changing from integrable to quantum chaotic pass through critical quantum chaos [7]–[10] which is also a metal-insulator transition from Anderson localization to extended wave functions. By extending the semiclassical theory of decoherence from chaotic [11]–[17] and integrable systems [18] to the transition region we show that coherence decay changes from exponential to power law behavior and coherence time is amplified exponentially from its environmentally determined value. We demonstrate on a ring of chromophores that coherence in the critical point decays with the same non-trivial power law as in the FMO complex experiment [5].

## Results

Quantum biology is dealing with open quantum systems closely coupled to their many degrees of freedom environment. The environment exerts time dependent forces on the system through the coupling. Some of these forces change very rapidly compared to the excitation frequencies of the system and look random from its point of view. This “heat bath” destroys quantum coherence and moves the system into a mixed state rapidly. The average effect of the random forces can be described as a non-unitary time evolution of the system's density matrix.

The speed of environmental decoherence can be characterized by the decay rate of the off diagonal (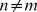) elements of the reduced density matrix of the system 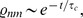 where 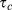 is the coherence time. Purity 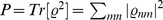 has been shown to be a good overall measure. It is 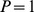 when the system is in a pure state and decreases monotonically as the system decoheres into a mixed state. 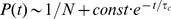, where 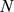 is the number of quantum states of the system. The logarithm of the purity is the Renyi entropy 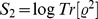 of the system. The long time entropy production rate of the system [12] and the rate of decoherence are then closely related 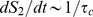 for 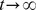. Entropy production on the other hand is determined by the dynamical properties of the system. It has been derived via semiclassical approximation and then proven by direct simulations that the entropy production rate becomes environment independent and is determined by the classical dynamical Kolmogorov-Sinai entropy of the system [11]–[17]. It is in turn the sum of the positive Lyapunov exponents 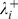 characterizing the exponential divergence of chaotic trajectories in the system 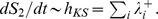 This relation between coherence decay and generalized Lyapunov exponents has been confirmed in strongly chaotic systems. Another implication of this result is that the rate of decoherence vanishes in systems where the Lyapunov exponent is zero. This has also been confirmed in integrable systems. These are completely solvable systems with fully predictable regular dynamics and zero Lyapunov exponents. Purity shows power law decay typically like 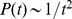 and asymptotic decoherence rate is zero 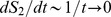.

Zero Lyapunov exponent and entropy production can also emerge in systems at the border of the onset of global chaos in the classical counterpart of the system. Suppose, we have a parameter 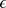 of the mechanical system which characterizes its transition from regular dynamics to chaos [20], [21] 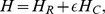 where 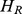 is the Hamiltonian of a fully integrable system and 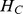 is fully chaotic. Classically 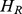 is a solvable system and it can be described by action-angle variables. It does only simple oscillations in the angle variables while the action variables do not change and remain conserved restricting the dynamics for the surface of a torus in the phase-space. When 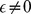 but small the system is not integrable classically and the Kolmogorov-Arnold-Moser (KAM) theory describes the system [20], [21]. The chaotic perturbation breaks up some of the regular tori in the phase-space and chaotic diffusion emerges localized between unbroken, so called KAM tori. Chaotic regions are localized in small patches in the phase-space surrounded by regular boundaries represented by the KAM tori. At a given critical 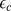 even the last KAM tori separating the system gets broken and the chaotic patches merge into a single extended chaotic sea. In the transition region 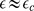 the Lyapunov exponent shows a second order phase transition [22] with power law scaling 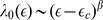 slightly above 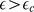 with some exponent 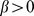. Above the transition 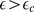 the system is chaotic characterized by a positive largest Lyapunov exponent 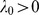.

On the quantum mechanical level we can follow the transition in the statistical distribution of the energy levels. The Hamilton operator of the regular system 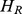 is a separable with diagonal matrix elements. The consecutive energy levels of the regular system look random and follow a Poisson process. The nearest neighbor level spacing distribution is then exponential 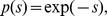 where 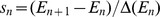 is the level spacing measured in the units of local mean level spacing 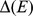 at energy 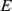. The Hamiltonian operator 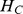 corresponding to the fully chaotic system is best approximated by a random matrix. The energy level statistics of 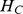 can be described by Random Matrix Theory (RMT) and the level spacings follow the Wigner level spacing distribution [23] 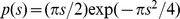 in systems with time reversal symmetry. As the parameter 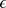 is increased from zero the level spacing statistics changes from a Poissonian to a Wigner distribution. Critical quantum chaos [10] sets up at the critical value 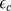 in between. Below the critical point 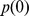 is finite, at the critical point and above the spacing distribution starts linearly 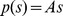 for 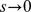, a characteristic feature of chaotic systems with strongly overlapping eigenfunctions. The tail of the distribution remains exponential below the critical point 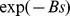 for 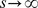 which is a characteristics of regular systems whose eigenfunctions do not overlap for larger energy separations. It turns to gaussian 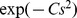 then above the critical point. At the critical point the level statistics is semi-Poisson [10] 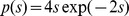 which starts linearly and decays exponentially combining the two main aspects of the level statistics of regular and chaotic systems.

The transition described here is more general than just the transition from regular to quantum chaotic behavior. It is also a transition from the localized states of the regular system to the extended states of the chaotic system. The two are separated by the metal-insulator transition (MIT) point [7], [9] between quantum mechanical Anderson localization and globally delocalized metallic phase. The transition point can be identified with the emergence of the semi-Poissonian [9] level statistics. In the transition point the wave functions are neither fully localized nor extended and have an intriguing multi-fractal spatial character. The fractal structure allows them to develop a hairy localized structure but also an extended structure with long range overlap correlations.

Merging the pieces of classical, semiclassical and quantum aspects a new picture emerges. Systems well below the critical point have non-chaotic dynamics with zero generalized Lyapunov exponents and quantum localization lengths extending only for few states. Decoherence in these systems is slow and purity follows a power law decay 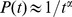 with some exponent 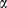 making possible the presence of anomalously long living coherent dynamics in the system. But coherently evolving states remain localized and long distance quantum coherent transport is not possible. Systems well above the critical point have chaotic dynamics with positive Lyapunov exponents and delocalized states extending for the entire system. Coherence dies out exponentially fast. Near the critical point exponential decay of coherence crosses over to long living power law behavior and localized states become delocalized. In finite systems there is always a narrow region around criticality, where long living coherence and sufficiently extended states can exist at the same time.

We demonstrate this on a simplified model of chromophores in light harvesting complexes and argue that it is very likely that biological systems use this mechanism to tune their parameters [24] near the critical point to maintain rich quantum transport properties. The excitonic states are described in the single excitation approximation by the Hamiltonian 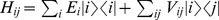, where 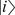 indexes the excitonic states with site energies 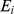 and dipole interaction strengths 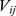. For simplicity we take a simple ring of 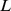 chromophores coupled by constant 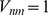 for neighboring sites 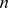 and 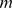 and zero otherwise and take quasi random on site energies given by 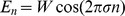, where the irrational number 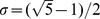 is the golden mean. This hamiltonian is known as the one dimensional Harper model. At 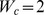 the infinite system 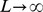 goes through a MIT with delocalized states below and localized states above criticality. At the critical point it has been shown to have semi-Poisson level statistics [27]. The system is coupled to the phononic environment via the Hamiltonian 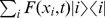, where 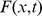 is the randomly fluctuating phonon field including the chromophore site energy coupling constant. The reduced density matrix of the chromophore system can be described in Markovian approximation by the Lindblad equation [26]

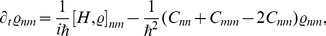
(1)where 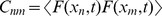 is the correlation function of the environmental coupling. We assume that the correlation function depends only on the periodic distance of the chromophores in a simple way 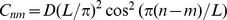 and is quadratic for small distances.

**Figure 1:**
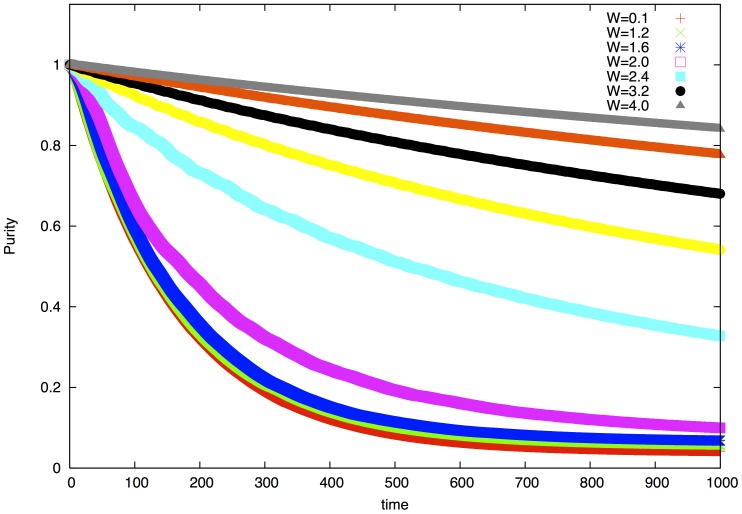
Purity decay of the chromophore ring with 1D Harper hamiltonian. Below the metal-insulator transition 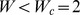 curves can be fitted with exponentials 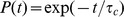. In the parameter range 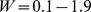 the fitted coherence time changes in the range 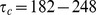. In and above the transition 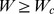 the curves can be fitted with 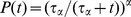. In the parameter range 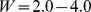 the exponent changes in the range 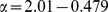 and 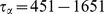. The estimate for the decoherence time 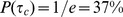 above the transition is 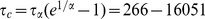.

**Figure 2:**
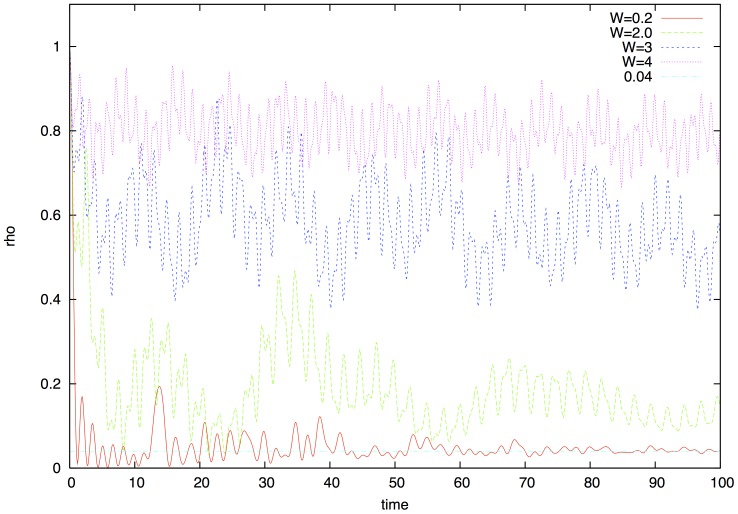
Probability that the exciton stays on the chromophore it started in represented by the density matrix element 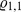. Below the transition (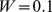) coherence dies out quickly and the probability reaches its asymptotic value 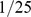. The peak at 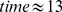 is the result of the interference of waves going clock and anti-clockwise along the the circle and meeting again after turning around the structure. The rest of the structure comes from interference of waves scattering back from other chromophores. In the transition point and above coherent beats occur and the probability stays elevated for a very long time (not shown here). These are beats due to genuine quantum coherent superposition states.

**Figure 3:**
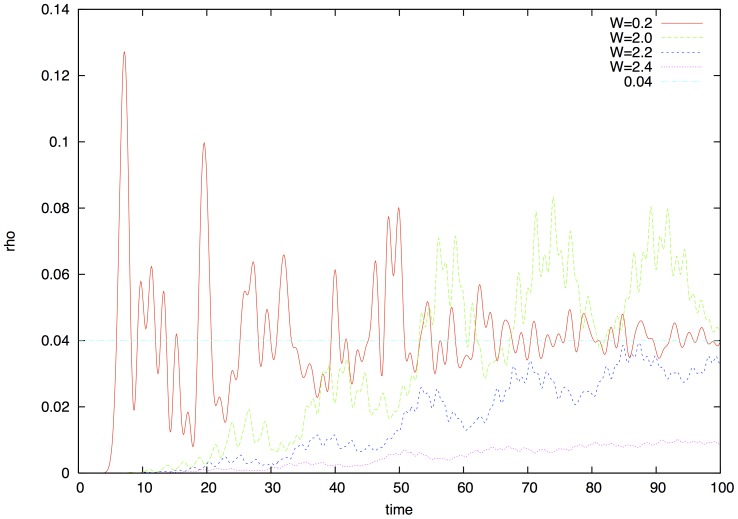
Probability that the exciton started on chromophore 1 is on chromophore 13 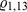. Below the transition (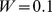) coherence dies out quickly and the probability reaches its asymptotic value 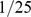. The beats at times 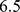 and at 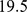 reflect interference from clock and anti-clockwise traveling waves interfering after taking half and 1.5 rounds. In the transition point and above we can see that due to the localization it takes much longer time for the excitation to arrive at the opposite end. If we are just slightly above the transition coherent beats smear out by the time they arrive. The reason is that propagation becomes mostly classical as localization stops quantum diffusion. In the critical point however quantum propagation and coherence is possible at the same time.

Next we show results for 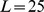 (in dimensionless units 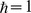), which is a realistic number of chromophores in experimentally investigated systems [25]. In Fig. 1 we show purity of the system. Below the critical point purity decays exponentially. At and above the critical point the curves can be fitted with power law exponents changing from 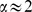 at criticality towards zero as 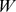 increases and the curves flatten out. In Fig. 2 we show the probability 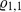 to find the exciton on the chromophore in which the exciton was initialized. Below criticality the probability reaches its asymptotic value of 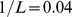 quickly after decaying coherent oscillations. Above criticality the probability stays above the asymptotic value for a long time indicating the presence of localization. Quantum beats can be observed which also relax in a very slow fashion. Based on the simulations we can establish the rule of localization assisted amplification of coherence time. In the delocalized regime purity decays exponentially determined by the timescale dictated by the environment. In the localized regime we can define an effective coherence time by looking at the point where purity decays to 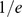 of its original value 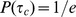. Above the critical point purity can be well approximated by function 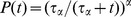 (see Fig. 1). The effective length of coherence then can be approximated as 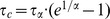. This function grows very fast when 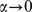 in the strongly localized limit. In our example this is a 60-fold increase between criticality and 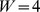. In Fig. 3 we show the probability 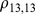 of finding the excitation at the opposite end of the ring. For subcritical values the excitation arrives very quickly due to delocalization and shows beats due to the interference of excitons traveling clock and anti-clockwise. Coherent beats die out quickly and we reach the asymptotic probability. For supra-critical values far from the critical point the probability to reach site 13 remains astonishingly low due to the localization of the system. For values near at and below criticality we get the most optimal results for quantum coherent transport of excitations, when excitations can still reach the opposite end of the circle but can preserve a degree of coherence as well.

**Figure 4:**
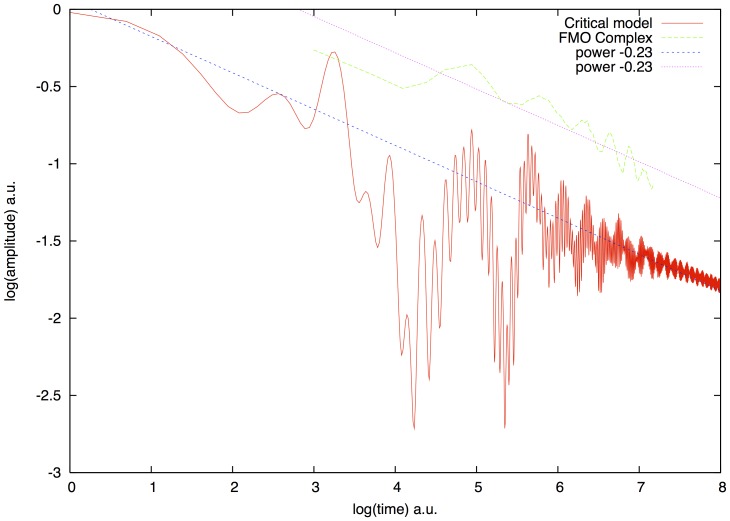
Power law decay of the population probability 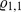 for the chromophore model and for the real FMO complex. The population decay for a modified version of our model is shown (red) on log-log scale at the critical point 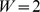, where each site is fully correlated with its next neighbors and not correlated with the rest of the chromophores. The same quantity is shown for the FMO complex (green). Time scales are in arbitrary units. Both curves oscillate around a trend which decays with the same exponent of about 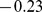. While the geometry is different for FMO and the ring, both of them seem to follow the same, perhaps universal, scale free trend observed at the critical point of the MIT.

At criticality not only purity changes from exponential to power law decay but so does the population of the chromophores. In Fig. 4 we show the population 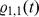 in a version of our model where three neighboring chromophores along the circle are always fully correlated 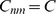 while they become totally uncorrelated otherwise 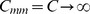. This model can describe the real situation where neighboring chromophores are shielded from the environment by their protein scaffolds and approximately three chromophores can be placed within the protected thermal wavelength of 10–13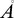. We can see that the trend of the population follows a shallow power law decay. We show also the experimental data of Ref. 5 on the FMO light harvesting complex kindly provided by the authors. Both curves follow a similar scale free trend with approximately the same exponent 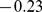. This exponent is very close to 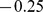 which is the power law decay exponent of the average return probability 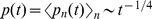 at the critical point of MIT as it was shown in Ref. [28]. The return probability 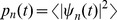 is the probability of return assuming that the wave function was localized on the site initially 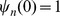. In the decoherence free case it coincides with the density matrix element 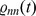 assuming 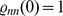, which is shown in our model and for the FMO complex. It seems likely that the FMO complex follows the universal scaling of critical MIT indicating that the hamiltonian of the FMO complex is tuned to critical quantum chaos in order to realize optimal coherent transport, what we show elsewhere.

## Discussion

The findings support a new approach to quantum biological systems. They are not just under the influence of environmental decoherence due to random noise but also driven by waves of the incoming photons. The photons are absorbed by the chromophores which initiates an exciton on one of the chromophores in an initial state which is concentrated on the selected chromophore. The purity of the system becomes 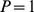. Then the partially decoherent evolution starts again decreasing the purity in time and the system can hover in the “Poised Realm” [19] between clean quantum and incoherent classical worlds. By tuning the timings of re-coherence events and the coherence time during decoherence via tuning the system on the chaos-regularity axis can be kept in high level of purity. This makes it possible to create new quantum devices working at room temperature capable of nearly frictionless quantum transport of energy and information.
